# Correlation between Magnetic Properties and Chemical Composition of Non-Oriented Electrical Steels Cut through Different Technologies

**DOI:** 10.3390/ma13061455

**Published:** 2020-03-23

**Authors:** Gheorghe Paltanea, Veronica Manescu (Paltanea), Radu Stefanoiu, Iosif Vasile Nemoianu, Horia Gavrila

**Affiliations:** 1 Faculty of Electrical Engineering, University Politehnica of Bucharest, 313 Splaiul Independentei, District 6, RO-060042 Bucharest, Romania; gheorghe.paltanea@upb.ro (G.P.); iosif.nemoianu@upb.ro (I.V.N.); horia.gavrila@upb.ro (H.G.); 2 Faculty of Material Science & Engineering, University Politehnica of Bucharest, 313 Splaiul Independentei, District 6, RO-060042 Bucharest, Romania; radu.stefanoiu@upb.ro

**Keywords:** soft magnetic materials, non-oriented electrical steels, energy loss separation, cutting technology, magnetic permeability, chemical composition, grain size

## Abstract

Due to worldwide regulations on electric motor manufacturing, the energy efficiency of these devices has to be constantly improved. A solution may reside in the fact that high quality materials and adequate cutting technologies should be carefully chosen. The magnetic properties of non-oriented electrical steels are affected by the cutting methods, through induced plastic, and thermal stresses. There is also an important correlation between chemical composition and different magnetic properties. In this paper, we analyze different industrial grades of non-oriented electrical steels, used in electrical machines’ core manufacturing as M800-65A, M800-50A, M400-65A, M400-50A, M300-35A, and NO20. The influence of the cutting methods on the normal magnetization curve, total energy loss and its components, and relative magnetic permeability is investigated in alternating currents using a laboratory single sheet tester. The chemical composition and grain size influence are analyzed and correlated with the magnetic properties. Special attention is devoted to the influence of the increased cutting perimeter on the energy losses and to the way it relates to each chemical alloy constituent. The final decision in what concerns the choice of the proper magnetic material and the specific cutting technology for the motor magnetic cores is imposed by the desired efficiency class and the specific industrial applications.

## 1. Introduction

Nowadays, the European Union (EU) firmly sustains energy efficiency policies through different programs such as Eco-Design and Energy Efficiency Directive, usually in conjunction with climate policies, such as the EU Emission Trading scheme. The main expected outcome is the improvement of energy efficiency for all the equipment in the industrial sector [1,2,3]. The greenhouse gas emission must be reduced by 20% below the levels recorded in 1990. Furthermore, the energy produced, due to renewable sources has to be increased by 20% and motors’ energy efficiency must also increase by 20%, in order to supply the total energy consumption [4,5]. Thus, during the design of the manufacturing process of an electric motor, the producers should make a careful material selection, which is supposed to be one of the most important characteristics of the motor life cycle. There are preferred high and medium quality non-oriented electrical steels, with low impact on the environment [6,7,8]. Their chemical composition is very important, in order to assure superior magnetic and mechanical properties. The high-quality non-oriented electrical steels become important in the high efficiency motor manufacturing, electrical vehicle production and power generation installations.

Non-oriented electrical steels have excellent magnetic properties that are obtained by cold-rolling intermediate manufacturing steps [9,10], but their energy loss and magnetic permeability are strongly influenced by the strip cutting technology and by the motor core forming procedure [11,12]. The electrical steel has structural changes due to machining and cold-working procedures; they can be deeply investigated with magnetic methods [13]. The most used and cheaper cutting procedure is the mechanical one. It is based on a shearing process that appears along the material, when it is plastically deformed. A negative effect, which consists of a strain hardening phenomenon, is put in evidence near the cutting edge. It is the reason why non-conventional cutting technologies are preferred, such as laser cutting, which generates small heat-affected zones (HAZ). The induced thermal stresses are directly linked to an increase of the energy loss and to a decrease of the magnetic permeability. Other non-conventional methods worth to be mentioned are water-jet and electroerosion technologies. In the case of the water-jet procedure, a high-pressure water-jet, in which abrasive particle are added, cuts the material through a rapid erosion phenomenon. Concerning the electroerosion method, an electro discharge machine (EDM) or a wire combined with an EDM (WEDM) are used. The WEDM technology is based on an electrode presence, which initializes the sparking procedure. Furthermore, it could involve a tensioned travelling wire, which erodes the material, with no contact between the wire and steels, so that the induced mechanical stresses are minimized. Due to their slow cutting speed, these technologies are predilectly used in the motor prototype manufacturing.

The present paper is devoted to analyzing the influence of the cutting technology on the magnetic properties of the alloys and correlating them with the chemical composition determination using optical emission spectrometry method, in order to highlight the cutting method side effects.

A first study was performed on M800-65A and M400-65A industrial grade steels from Surahammars Bruk AB, which were cut through mechanical punching, laser, water-jet, and electroerosion technologies, respectively. The samples were prepared as strips with a length of 300 mm and a width of 30 mm.

The second study investigates the impact of mechanical and water-jet cutting technologies on medium grade materials (M400-50A and M400-65A) but also on higher quality thinner grades steels (Co Gent NO20 and M300-35A). The cutting perimeter was increased on the same surface area of the tested sheets, in order to simulate a real situation existing in an electric motor, so that the samples were prepared with widths ranging from 5 mm to 60 mm at the same constant length that was kept at 300 mm.

In both cases, the normal magnetization curve, measured in quasistatic conditions, and the energy loss, at different peak magnetic polarizations *J*_p_, are determined and analyzed. The total energy loss is experimentally measured in a range of frequencies from 3 Hz to 400 Hz using a laboratory digital wattmeter. The statistical theory of loss [14] is applied to determine the hysteresis, classical (Foucault), and excess (anomalous) components.

The subject of work hardening by cutting has indeed attracted substantial efforts in the literature in recent years. These are investigations having a predominant phenomenological character [15,16,17,18,19,20,21,22]. They aim, on the one hand, at determining the extent of the structural damage, the residual stress, and the induction profile across the sample width using, in some cases, neutron grating interferometry. Moreover, they are generally directed towards relating the cutting methods and the corresponding parameters to the behavior of the total loss at power frequencies, with the eventual aim of introducing the results in the calculation of iron loss specific to rotating machines. However, the physical problem cannot be fully investigated, and simple predictions cannot be formulated without being inquisitive about the role and behavior of the loss components and their individual response to the cutting-induced strain hardening. Each component depends in a specific way on frequency and peak magnetic induction but also on sample geometry and material treatment. This kind of approach is conspicuously lacking in the literature. In the present paper we propose an approach to clarify this problem. The novelty of this work consists in the fact that the main industrial non-oriented alloys that are used in the core manufacture of the high-efficiency electrical machines are completely characterized from the viewpoint of the chemical composition and magnetic properties. The steels were cut through different methods, as mentioned above, in order to analyze the cutting technology influence on energy loss, normal magnetization curve, and relative magnetic permeability.

## 2. Materials and Methods

Two types of experimental studies were conducted in the paper. Samples from different industrial grades of non-oriented steels were prepared.

For the first type of analysis, M800-65A and M800-50A materials were chosen, some low-grade alloys, to further investigate M400-65A and M400-50A as medium quality grade steels. The 0.65 mm thickness samples (see Table 1) were cut at a width of 30 mm and a length of 300 mm, using mechanical punching, laser, water-jet and electroerosion technologies, respectively. The 0.50 mm thickness probes were prepared only using mechanical and water-jet methods. For the cutting procedure, we utilized the following equipment:

- In the case of mechanical cutting, a Krrass Q11-2X2500 electric shearing machine (Nanjing Klaus CNC Machinery Co., Ltd. (KRRASS), Nanjing, China) was used. The device is a steel-welded and gate-type with a chain drive and it is adequate to cut strips of metals having less than 3 mm in thickness [23].
- For the laser cut, a Trumpf TruFlow CO_2_ laser 3030 Classic (TRUMPF GmbH + Co. KG., Stuttgart, Germany) with a maximum power of 3200 W was used. The laser wavelength is 10.6 µm and the beam quality K 0.6. This machine has a correlation of 2% between beam stability and laser power. The generated cutting edge is extremely smooth and post-processing operations are not necessary. Usually, the final result is a micro-burr-free cutting sample [24].
- A Maxiem 1530 (OMAX Corp., Kent, WA, USA) with the water-jet supplied by a high-pressure water pump, which uses a mixture of Garnet 80 mesh, was selected to cut the samples. This machine is equipped with a linear motion system, based on linear encoders, in order to provide a high-quality cut edge [25].
- A Sodick Wire EDM VL600Q machine (Sodick Co., Ltd., Yokohama, Japan) was involved to cut the samples. This equipment has a rigid linear motor, which provides a good quality cut edge. It incorporates a ceramic material that has a high abrasion resistance and a low thermal expansion coefficient [26].

**Figure 1:**
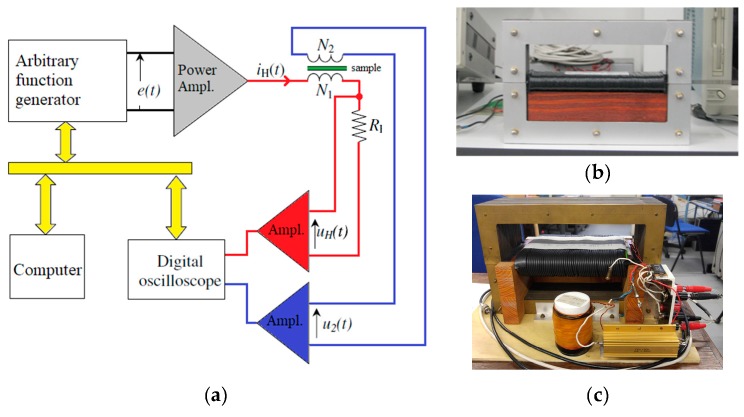
Digital wattmeter set-up (**a**) block-diagram [14], (**b**) first yoke for 30 × 300 mm^2^ samples, (**c**) second yoke for 60 × 300 mm^2^ samples.

The magnetic characterization of the samples was done utilizing a laboratory digital wattmeter. This device has two double-C laminated magnetic yokes. The one used in the first experimental study is made of 0.3 mm high-quality, grain-oriented silicon iron strips. The magnetic field coil has 173 turns and the equipment has a magnetic path length of 150 mm. The magnetic flux density measuring coil has 101 turns with a length of 20 mm. A 12-bit 500 MHz HDO4045 LeCroy oscilloscope (Teledyne LeCroy, Chestnut Ridge, NY, USA) was also used. The field coil was powered by a NF HSA4101 amplifier (NF Corp., Yokohama, Japan), which was controlled by an Agilent 33210A arbitrary function generator (Keysight, Santa Rosa, CA, USA) (see Figure 1). The first yoke (Figure 1b) permits the characterization of samples with an area of 30 mm × 300 mm.

Figure 1a depicts the measuring circuit diagram, in which the main idea is to impose a predetermined sinusoidal time variation of the magnetic polarization *J*, proportional to the secondary voltage *u*_2_(*t*) through a recursive technique. At the first step, the *e*(*t*) waveform is provided by the arbitrary function generator that has as output the *J*(*H*) dependence. The component *J*(*t*) of the hysteresis dependence is not sinusoidal, so that a new *i_H_*(*t*) waveform is computed and an updated *e*(*t*) function is given. The process is recursively controlled until the form factor criteria imposed on *J*(*t*) variation is met [14,27].

In order to determine the normal magnetization curves of the samples, the measuring frequency was set at 2 Hz, allowing us to measure the minor symmetrical hysteresis loops at different values of the peak magnetic polarization, namely *J_p_* ∈ {0.005 T, 0.01 T, 0.02 T, 0.05 T, 0.1 T, 0.2 T, 0.5 T, 0.75 T, 0.90 T, 1 T, 1.1 T, 1.2 T, 1.3 T, 1.4 T, 1.5 T, 1.6 T}. The maximum tip points of each hysteresis cycle were extracted and then the normal magnetization curve was obtained. The influence of the cutting technology on the energy loss and magnetic permeability was experimentally investigated, by performing measurements for two values of the peak magnetic polarization *J_p_* (of 0.5 T and of 1.0 T) at different measuring frequencies comprised between 3 Hz and 400 Hz.

**Figure 2:**
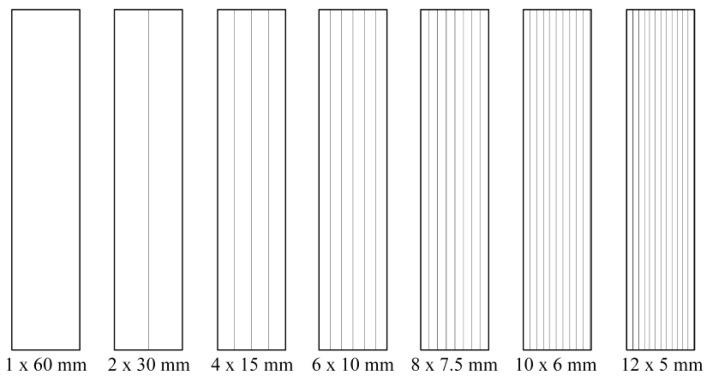
Strip assembly of the tested samples in the case of 60 mm × 300 mm total area.

For the second study, medium quality (M400-50A and M400-65A) and high quality (NO20 and M300-35A) steel samples were considered. The strips were cut through mechanical punching, using the Krrass Q11-2X2500 Machine, and trough water-jet technology, by means of a Maxiem 1530 device. The width *w* of the strip was chosen equal to 5, 6, 7.5, 10, 15, 30, 60 mm, respectively, and the length was kept constant at 300 mm. In order to reconstruct the width of 30 mm (for M400-50A and M400-65A) and of 60 mm (for NO20 and M300-35A), several identical strips were placed side by side, as shown in Figure 2. This procedure allows an accurate analysis of the influence presented by the cutting perimeter increase in which energy losses and magnetic permeability are concerned.

The 30 mm width samples were characterized by using the same digital wattmeter that was mentioned in the first experiment. For the 60 mm width samples, the yoke was replaced by other double-C laminated yoke that has a magnetic path length of 240 mm, a field coil of 288 turns and a measuring coil of 250 turns, as shown in Figure 1c.

All the measurements presented in the paper were conducted according to the IEC60404-3 standard [27]. For the second study, the increase of the cutting perimeter influence on the magnetic properties was investigated at a peak magnetic polarization *J_p_* of 1.0 T.

The physical and geometrical properties of the samples are presented in Table 1.

The medium grain size <*s*> for the materials, analyzed in the second study, was determined by means of an optical OM II Neophot 32 microscope. In order to prepare the samples for optical investigation, a small area was polished and etched by using a 5% Nital solution.

The chemical composition of the analyzed samples was determined by optical emission spectrometry using a spark optical emission spectrometer, SPECTROMAXx M type, with the following technical characteristics: Optical System Flat Field type, under vacuum, wavelengths between 130–780 nm, CCD detector solid DIC or CCS, excitation source type Spark stand closed, analytical performance—access to all spectra, without interruption [29].

## 3. Results

### 3.1. Chemical Composition of the Investigated Alloys

Decarburization, re-crystallization, and grain growth are the main steps in the electrical steel metallurgical generation process that is controlled by the chemical composition of the alloys, which is shown in Table 2.

The Si content is kept below 3.5%, in order to control the material brittleness, being aware that the step to final thickness implies a cold rolling process. The increase of Si content is directly linked to higher electrical resistivity from the low-grade M800-65A to the high-quality NO20 grade alloy, since a higher Si content has a beneficial influence on eddy current loss reduction and magnetostriction phenomenon limitation. The addition of Al determines an increase in electrical resistivity and grain size [30,31,32]. At the same time, aluminum oxide inclusions, which are relatively easily generated, create pinning sites that hinder the domain walls movement. Through the decarburization process, the C percent is limited because slow precipitation of carbides can determine an increase of hysteresis energy loss and of coercive field. By using a Mn addition, the austenitic structure of Fe is stabilized.

### 3.2. Influence of the Cutting Technology on the Normal Magnetization Curve, Energy Loss, and Magnetic Permeability Analysis

Usually, industrial applications of non-oriented electrical steels take into account the normal magnetization curve of the used materials. In order to measure the *J*(*H*) dependence, the magnetic field strength *H* is gradually increased starting from zero excitation. The experimental measurements were conducted as described in more detail throughout Section 2.

**Figure 3:**
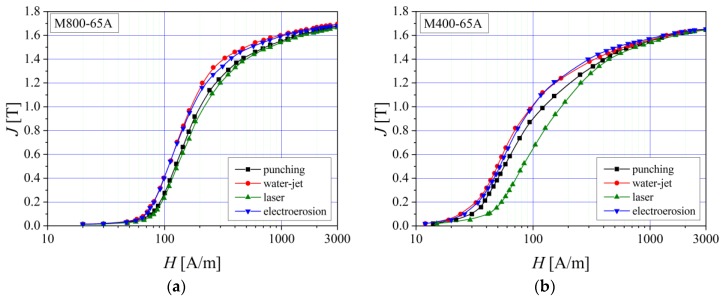
Normal magnetization curves measured at *f* = 2 Hz, in the case of (**a**) M800-65A and (**b**) M400-65A electrical steels.

It can be noticed from Figure 3 that the water-jet and electroerosion technologies lead to an easy magnetization process of the materials, because during the cutting procedure, the induced mechanical and thermal stresses both have a low value. In the case of M800-65A grade electrical steel, the influence of the cutting procedure on the normal magnetization curve is significantly reduced compared to the M400-65A behavior. This is due to the fact that M800-65A alloy has a higher number of magnetic impurities, which act as pinning sites for the magnetic domain walls movements. Consequently, in order to magnetize the material to saturation, it will be necessary to apply a higher magnetization field value. In both cases the laser and the mechanical punching are characterized by a much more difficult magnetization process, the effect being more pronounced in the case of the M400-65A grade alloy. The difference between the magnetization processes of M800-65A and M400-65A steels is due to their specific silicon content (2.2% for M400-65A and about 1.43% in the case of M800-65A). An important disadvantage could be considered the fact that the material becomes both hard and brittle, and the cutting technology influence on the normal magnetization curve increases, as in the case of M400-65A steel. The M400-65A alloy, which is a fully processed steel, has an average value of the grain size lower than 1 mm [33,34].

The influence of the steel thickness was analyzed in the case of M800 and M400 non-oriented alloys by comparing the 0.65 mm and the 0.50 mm thickness materials. As cutting methods, we considered the mechanical and water-jet cutting technologies, because punching is the most used technology by electric motors producers. Water-jet is adequate for prototyping manufacturing, as it induces lower stresses in the material.

**Figure 4:**
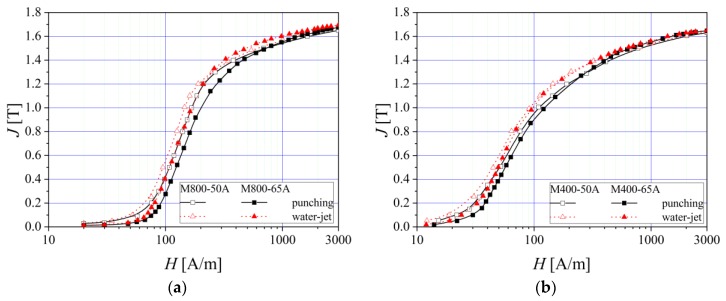
Normal magnetization curves of two electrical steel types (**a**) M800 and (**b**) M400, in the case of different sample thickness (0.50 mm and 0.65 mm).

From Figure 4, it can be observed that the water-jet cutting procedure leads to lower values of the applied field used to magnetize the samples, at the same value of the magnetic polarization as in the case of punched strips. The thickness decrease may conduct to a lower effect of the cutting technology on the magnetic properties. This fact is due to the reduced in-depth strain hardening effect influence on 0.50 mm thickness samples, in the case of punching technology.

**Figure 5:**
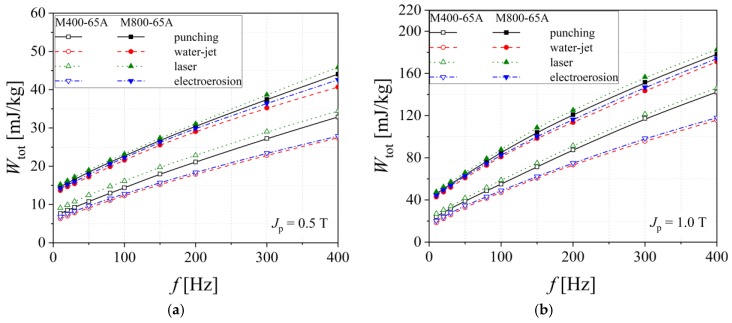
Total energy loss versus frequency measured at *J*_p_ = 0.5 T (**a**) and *J*_p_ = 1.0 T (**b**), in the case of M800-65A and M400-65A electrical steels.

The energy loss analysis as a function of frequency envisages a correlation between the alloy crystallographic texture and magnetization processes, giving a direct way for electrical machine producers to obtain energy efficient magnetic cores. As it was put in evidence in the case of the normal magnetization curve, the observation that the total energy loss in the case of M800-65A steel is not strongly influenced by the cutting technology, as compared to the M400-65A case, still remains valid. The explanation consists in the existence of larger non-magnetic impurities and low Si percent, as shown in Figure 5. For the M400-65A alloy, at the peak magnetic polarizations *J_p_* = 0.5, 1.0 T, the highest energy loss is put in evidence for laser and mechanical punching methods and shows the lowest value results in the case of water-jet technology. In the case of medium grade M400-65A steel, the total energy loss is divided into two categories as follows: the laser and the mechanical cutting lead to high energy loss, in the electroerosion case almost the same measured values for the energy loss as for the water-jet technology case are obtained. The increase of the Si and Al content, from 1.43% Si and 0.215% Al (in M800-65A steel) to 2.19% Si and 0.401% Al (for M400-65A), determines a decrease of the total energy loss.

**Figure 6:**
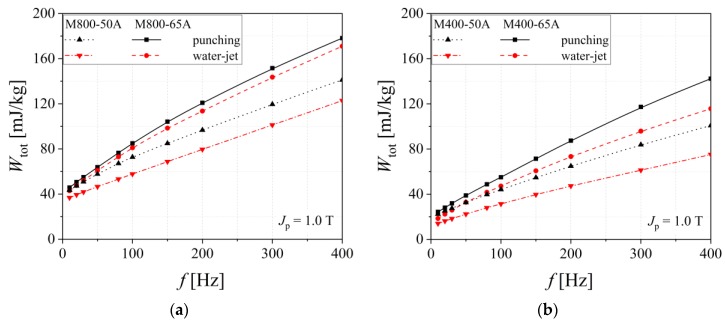
Total energy loss versus frequency measured at *J*_p_ = 1.0 T of two electrical steel types (**a**) M800 and (**b**) M400, in the case of different sample thickness (0.50 mm and 0.65 mm).

The influence of the sample thickness cut through mechanical punching and water-jet technologies on total energy loss is shown in Figure 4. In the case of M800 alloys, the probe thickness increase is linked to a more reduced cutting method influence on the energy loss (Figure 6a). For the M400 steels, the differences between loss figures, obtained for the punched and water-jet cut samples, are almost the same in the case of 0.65 mm and 0.50 mm thickness samples (see Figure 6b). In the case of both alloys, the 0.50 mm thickness steels exhibit lower energy loss.

**Figure 7:**
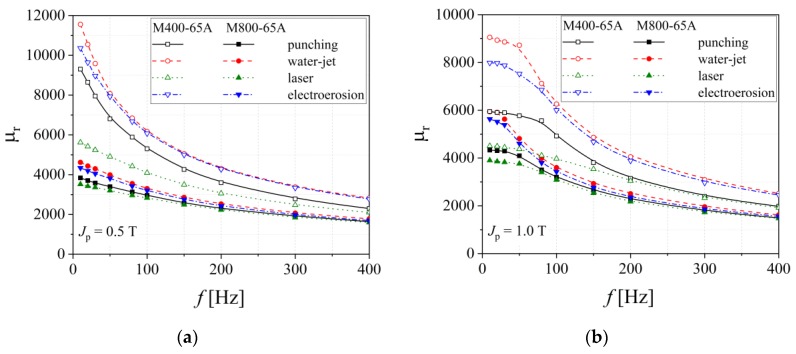
Relative magnetic permeability versus frequency measured at two values of *J*_p_ = 0.5 T (**a**) and 1.0 T (**b**), in the case of M800-65A and M400-65A electrical steels.

It is commonly accepted that an industrial grade with a medium silicon content (M400-65A) and with low energy loss has a lower coercive force and saturation polarization and a higher relative magnetic permeability. These alloys exhibit an important anisotropy of magnetic permeability, the highest values being obtained if the exciting magnetic field is applied parallel to the rolling direction [35]. The high magnetic permeability materials could be obtained, by slightly modifying the metallurgical process, by controlling the so-called dilution factor between magnetic alloy and austenitic phase steel (non-magnetic material) and by maximizing the average value of the permeability in the working zone of the device [35,36,37]. In the case of both analyzed non-oriented electrical steels, the relative magnetic permeability has a descending variation as the measuring frequency increases (Figure 7). Higher values are obtained for M400-65A alloy in the case of water-jet technology. It can be noticed that for the M800-65A grade, the cutting technology has an important influence at frequencies lower than 200 Hz, below these values the cutting technology presents a minimal effect.

**Figure 8:**
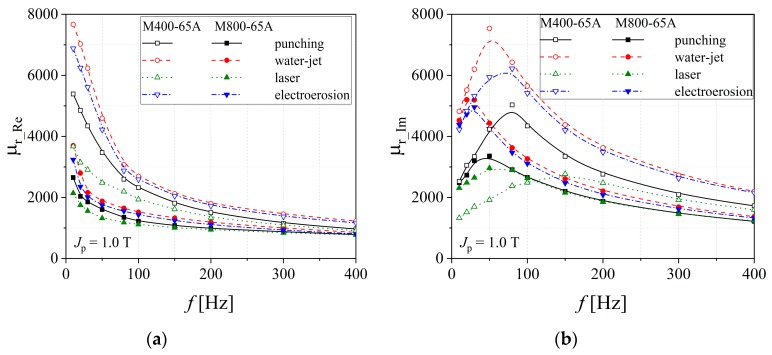
Real (**a**) and imaginary (**b**) components of relative magnetic permeability versus frequency measured at *J*_p_ = 1.0 T, in the case of M800-65A and M400-65A electrical steels.

The real part of the magnetic permeability is directly linked to the alloy capacity to channel the magnetic flux density lines within the material, in order to accumulate magnetic energy, whereas the imaginary part is usually associated with the energy loss. The real part of the magnetic permeability has a descending variation with frequency, while the imaginary part presents a maximum value (see Figure 8), which accounts for the relaxation phenomena existing within the various kinds of steel. These are due to the slow advancement of magnetic domain walls, which are pinned by the non-magnetic impurities.

### 3.3. Influence of the Icreased Cutting Density on the Energy Losses and Magnetic Permeability Analysis

The unwanted effect of mechanical stresses and strain-hardening phenomenon induced by punching produces an increase in total energy loss. The magnetic properties and the crystalline texture of the fully processed steel sheets that have been adjusted through different metallurgical treatments, are affected by the material processing when it comes to obtain the required geometries specific to the electrical machine magnetic core. The cutting, stacking, or assembly process should be taken into account when a rotor or a stator is built, through the induced detrimental effects on the magnetic properties. The influence of cutting on the electrical steels can be better investigated by reducing the width of the samples, in order to modify the residual stresses induced by different cutting procedures, as presented in Section 2.

**Figure 9:**
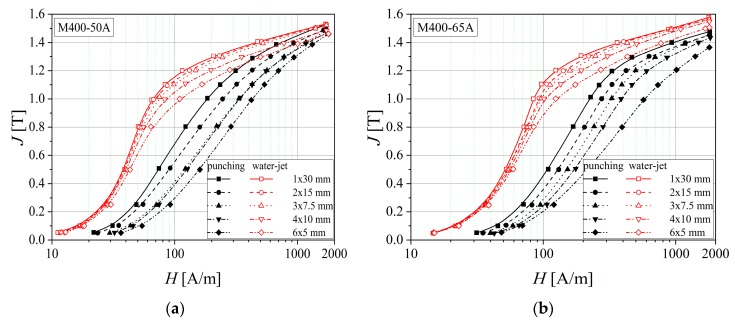
Normal magnetization curves of M400 electrical steel grade, in the case of different sample thickness (0.50 mm (**a**) and 0.65 mm (**b**)) and two cutting technologies.

Figure 9 shows that by decreasing the analyzed strip width, the magnetization process becomes increasingly difficult. For the water-jet cutting, the experimentally obtained normal magnetization curves show that lower values of the excitation field are required than those necessary in the case of punching technology for obtaining the same magnetic polarization value. At the saturation point, the reduced thickness (0.50 mm) of the samples results in an overlapping of the normal curves.

**Figure 10:**
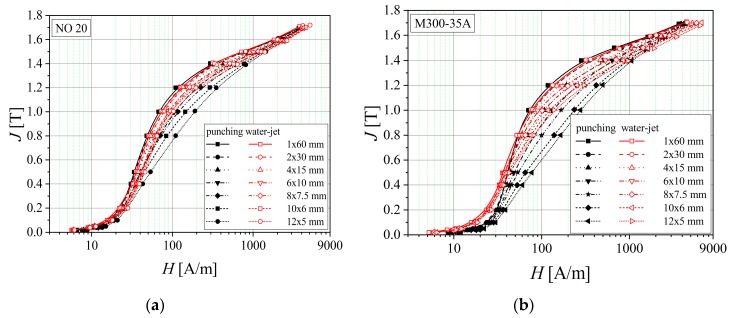
Normal magnetization curves of electrical steel grade NO20 (**a**) and M300-35A (**b**), in the case of two cutting technologies.

Figure 10 shows the normal magnetization curves as a function of strip width for NO20 and M300-35A samples. These electrical steels have a lower thickness than those of M400-65A and M400-50A, and the two analyzed cutting methods have a much more reduced impact among different samples. The 60 mm width sample has approximately the same magnetic behavior in the case of both cutting procedures, which indicates that the punching and water-jet methods have no influence on the alloy’s magnetic properties. Along with the sample width decrease, the magnetization processes become more difficult, because of the gradually increased number of cutting edges. The strain-hardening phenomenon that appears at the cut edges is reduced in the case of water-jet technology, since mechanical cutting affects a wider area and this technique changes the magnetic domain pattern [38]. The saturation magnetic polarization decreases with the silicon percent increase. In NO20 alloy, it was found to be 2.31%, in comparison with 1.95%, specific to M300-35A. Furthermore, it can be noticed that the mechanical cutting method is directly linked to a reduced saturation value, in the case of both materials, compared to the water cutting procedure, which exhibits a very limited influence.

**Figure 11:**
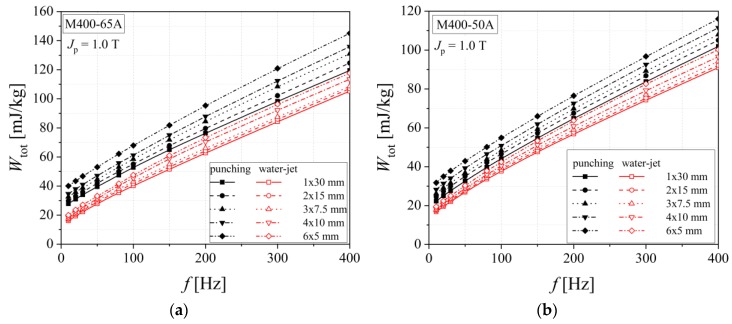
Total energy loss versus frequency dependencies of electrical steel grade M400-65A (**a**) and M400-50A (**b**), in the case of two cutting technologies.

**Figure 12:**
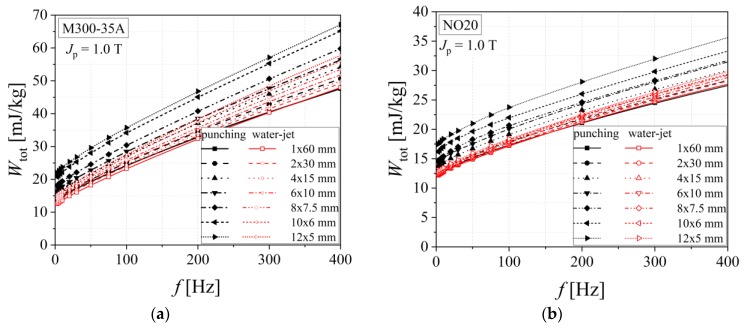
Total energy loss versus frequency dependencies of electrical steel grade NO20 (**a**) and M300-35A (**b**), in the case of two cutting technologies.

Figure 11 and Figure 12 show how the analyzed alloy quality influences the total energy loss that exhibits a decreasing evolution from medium grades to high quality steels. The increased cutting density determines, in the case of both procedures, higher values of total energy loss, with even a more pronounced impact in the case of guillotine cutting. According to Table 3, in the case of punching, there is a 20%–30% increase, and for the water-jet of just 5%–15%, in what concerns the total energy loss measured for the two extreme widths (*w* = 5 mm and 30 mm).

An increased Al content leads to a reduction of the classical energy loss. This result is explained through the Al effect on the electrical resistivity. The M300-35A alloy has the highest Al concentration (0.45%) and it exhibits good magnetic properties along the rolling direction. The Ti percent is very low in the investigated steels (between 0.003% in the case of NO20 and 0.008% for M300-35A). Titanium combines with C and N resulting in stable titanium carbonitrides, which favor the crystallographic texture development, but hinder the domain wall motion [39].

**Figure 13:**
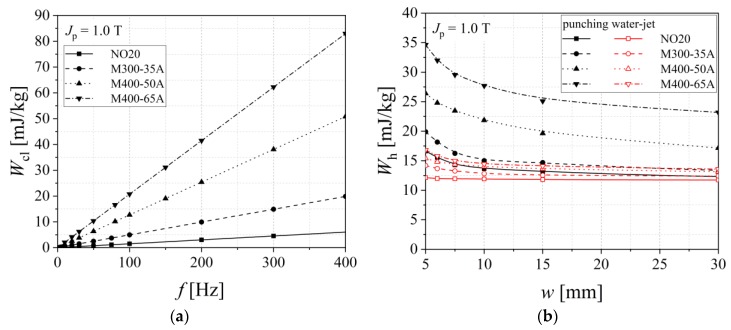
Classical (**a**) and hysteresis energy loss (**b**), in the case of two cutting technologies.

The total energy loss was analyzed according to the statistical theory of losses, which considers its decomposition into classical (eddy currents), hysteresis, and excess loss components [14]. The classical energy loss *W*_cl_ is associated with the macroscopic eddy currents, which are present in the close proximity of the sheet surface. Eddy current loss is computed on the basis of the strip thickness, resistivity, and density, being directly proportional to the working frequency at a given magnetic polarization value [40]. Under these assumptions, the classical loss may be considered as invariable with regards to the cutting technology, as shown in Figure 13a. In the case of electrical steels, the C content has to be limited to 0.01%, so as not to favor the slow precipitation of iron carbides, which leads to higher energy loss. The iron carbides determine a hysteresis loss increase and a reduction of the relative permeability. The decarburization treatment induces a diminution of the C content and a decrease of the residual stresses through the generation of an oxide layer on the alloy surface. A secondary re-crystallization, combined with grain size growth, leads to a better crystallographic texture. Sulfur is usually considered an impurity in the electrical steel manufacturing process, because during solidification, S reacts with Mn, forming inclusions of manganese sulfide (MnS). A S decrease content determines lower energy losses. The investigated alloys have the S percentage comprised between 0.003% (M400-65A, M400-50A) and 0.004% (M300-35A, NO20). Nitrogen reacts with Al and the resulted AlN particles, which as well as MnS, hinder the magnetic domain wall movement. If they are thin, an inhibition of the grain growth is observed, during the electrical steel decarburization step [41,42]. The hysteresis energy loss (see Figure 13b) was computed by extrapolating the total energy loss values as a function of frequency toward zero. The highest hysteresis loss was determined in the case of M400-65A, for the punched 5 mm width strip, being equal to 35 mJ/kg, whereas the lowest value of 12 mJ/kg was found in the case of the NO20 water cut 60 mm width sample.

The excess (anomalous) energy losses is directly linked to the domain wall motion and it is influenced by the grain size and the crystallographic texture. The Si and P percent has an important effect on the excess loss, by increasing the grain size [43]. It is well known that the main contribution to the total energy loss is accountable to the hysteresis loss component, but the existence of the precipitated impurities’ content or larger grain size makes the excess loss to become important. According to Bertotti’s theory [14,44] the excess energy loss is governed by the competition between the action of the externally applied magnetic field and the spatial variations of the local coercive fields, which are due to the Bloch walls’ existence. The character of these fluctuations is correlated, in non-oriented steels, to the grain dimension, rendering the average grain size to become very important [44].

**Figure 14:**
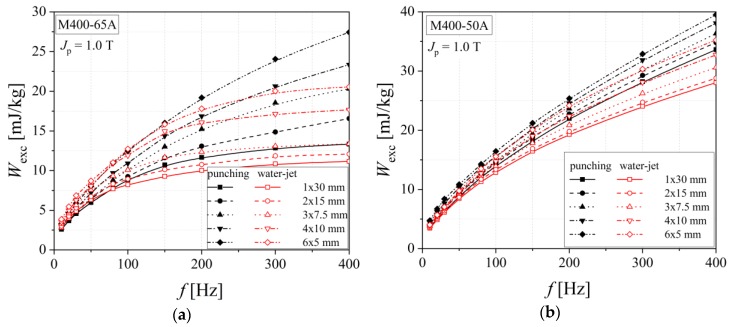
Excess energy loss versus frequency dependencies of electrical steel grade M400-65A (**a**) and M400-50A (**b**), in the case of two cutting technologies.

The average grain size <*s*> was determined through optical microscopy method, shown in Table 4:

The influence of the cutting procedure on the dynamic energy loss, that represents the sum of classical and excess components in the 3 Hz–400 Hz frequency range, is put in evidence by representing the excess loss component versus frequency, in good accordance with the statistical theory of losses. This links the strain hardening effect generated by cutting to the statistics of the local coercive fields, and the final result is an increased value of excess loss. In the case of medium quality M400-65 alloy, the excess loss is lower than that obtained for M400-50A steel, because of the larger grain size and the higher Si content (2.19% for the 65 mm and 1.99% in the case of 50 mm thickness alloy) (see Figure 14). The P content ratio was kept to 0.021% for both materials.

**Figure 15:**
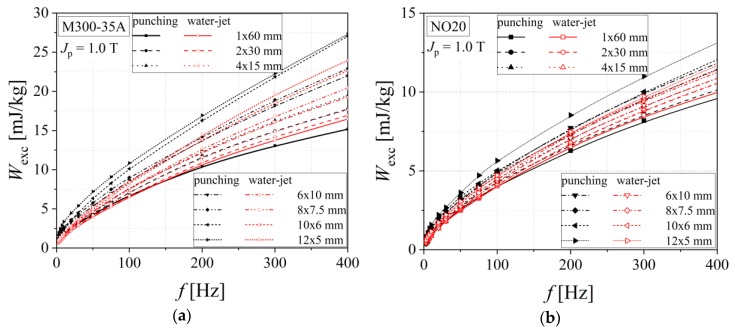
Excess energy loss versus frequency dependencies of electrical steel grade M300-35A (**a**) and NO20 (**b**), in the case of two cutting technologies.

Figure 15 shows the excess energy loss in the case of the investigated high-quality electrical steels. It can be noticed that the anomalous loss increases by increasing of the cutting density. This phenomenon is accountable to the fact that magnetic hardness caused by plastic deformation becomes stronger for the smaller strip width. The dependencies obtained for water cut samples are included between the two limit curves, namely the ones determined for the 60 mm and 5 mm widths in the case of mechanically cut samples. The excess energy loss presents higher values for the M300-35A alloy than for NO20, for both cutting technologies. This observation is in a good accordance with the fact that NO20 steel has a larger grain size. The M300-35 A steel has a lower percent of Si (1.95%) and P (0.029%) than NO20 alloy (Si 2.31% and P 0.033%), thereby an increased excess loss is perfectly justified.

**Figure 16:**
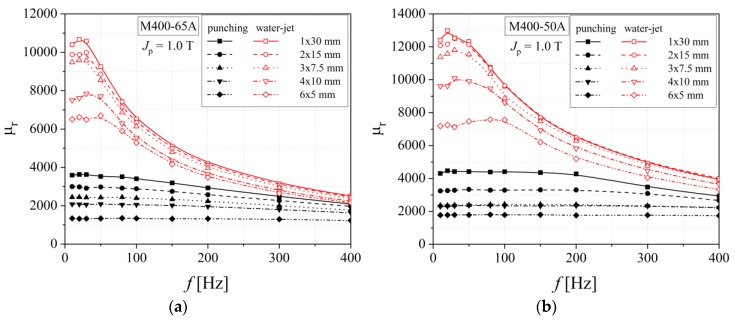
Relative magnetic permeability versus frequency dependencies of electrical steel grade M400-65A (**a**) and M400-50A (**b**), in the case of two cutting technologies.

**Figure 17:**
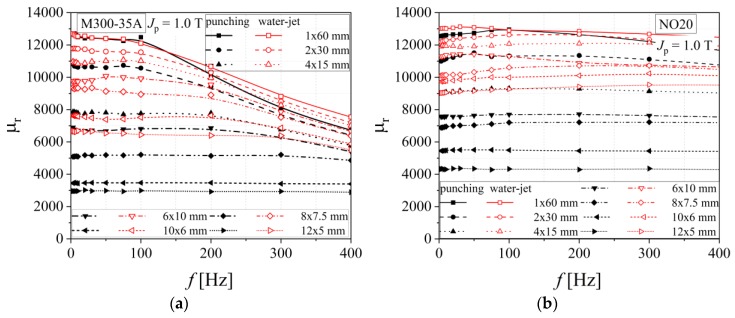
Relative magnetic permeability versus frequency dependencies of electrical steel grade M300-35A (**a**) and NO20 (**b**), in the case of two cutting technologies.

The water-jet method has lower influence on the relative magnetic permeability than the classical mechanical cutting in the case of all investigated steels, as shown in Figure 16 and Figure 17. In the case of M400 alloys, a clear separation between relative permeability curves, obtained in the case of punching and water-jet technologies, is present, when compared with the thinner M300 and NO20 steels. This fact can be correlated with the increased strain hardening phenomena that appears during mechanical cutting for increased sample thickness.

The width of the damaged zone was estimated to be between (3.5 ÷ 4.2) mm in the case of punching and (3 ÷ 3.4) mm for water-jet cut method [45,46,47], with higher values in the case of M400 steels, fact which determines a decrease of the relative magnetic permeability modulus.

## 4. Conclusions

In the paper we have analyzed the magnetic behavior of industrial medium- and high-quality non-oriented alloys, cut through different technologies. We have focused on the evolution of the normal magnetization curve, total energy loss, and its components (hysteresis, classical, and excess loss) and relative magnetic permeability. The influence of the plastic and thermal stresses, induced by cutting technologies, was correlated with the chemical composition and with the degradation of the soft magnetic material behavior, consisting in an increase of the energy loss and a decrease of the magnetic permeability. The main conclusion of our study is that the physical properties improvement of magnetic alloys used in electrical machines manufacturing results as a balanced solution, in which several critical factors are involved: chemical composition, fabrication, and sheet cutting technologies. To summarize, the water-jet cutting technology provides electrical steel samples with best preserved characteristics, in terms of energy loss and relative magnetic permeability. Unfortunately, this method cannot be implemented on a larger-scale manufacturing process of electrical machines, because this process is too slow. Opposite to the water-jet technology, the most detrimental effect on the energy losses and relative magnetic permeability is due to the laser cutting. The electroerosion method has also a reduced influence on the magnetic properties, while the mechanical cutting increases the energy loss even more but still remains the classical and most preferred technology for motor manufacturers. The extend of the magnetic properties’ deterioration, due to cutting procedures can be emphasized by analyzing the energy loss values. If we consider the M400-65A electrical steel, at peak magnetic polarization of 1 T and measuring frequency of 50 Hz, the total energy loss in the case of the water-jet cut sample is 32.88 mJ/kg and for the laser cut strips it increases to 41.79 mJ/kg. The chemical composition of the alloy is a key element, because by slightly modifying its constituents, the total energy loss could be reduced, and the magnetic permeability could be increased. The number of impurities is different in the case of medium- and high-quality steels, due to the formation of chemical precipitates, e.g., MnS and AlN or carbonitrides that reduce grain growth and detrimentally influences material texture, and hence this phenomenon has to be limited. The addition of Si is a very important factor, therefore, an optimal silicon percent should be carefully chosen for each specific device under development and design.

The final decision when it comes to choose the proper magnetic material and its specific cutting technology for the motor magnetic cores is dictated by the efficiency class design specifications along with the particularities of the industrial applications themselves.

## Figures and Tables

**Table 1:** Physical and geometrical properties of the investigated non-oriented electrical steels [28].

Sample Grade	Mass Density [g/cm^3^]	Electrical Resistivity [Ωm]	Thickness [mm]
M800-65A	7.80	25 × 10^−8^	0.65
M800-50A	7.80	23 × 10^−8^	0.50
M400-65A	7.65	44 × 10^−8^	0.65
M400-50A	7.70	42 × 10^−8^	0.50
M300-35A	7.65	50 × 10^−8^	0.35
NO20	7.65	52 × 10^−8^	0.20

**Table 2:** Chemical composition of non-oriented electrical steels.

Sample Grade	Chemical Element						
	Fe [%]	Si [%]	Mn [%]	Al [%]	P [%]	S [%]	C [%]
M800-65A	96.4	1.43	0.58	0.215	0.049	0.005	0.033
M800-50A	98.3	1.177	0.213	0.129	0.043	0.005	0.0095
M400-65A	96.8	2.19	0.146	0.401	0.021	0.003	0.0095
M400-50A	97.1	1.99	0.183	0.376	0.021	0.003	0.0094
M300-35A	96.7	1.95	0.251	0.458	0.029	0.004	0.0094
NO20	96.5	2.31	0.185	0.341	0.033	0.004	0.0120

**Table 3:** Total energy loss *W*_tot_ [mJ/kg] at frequency *f* = 50 Hz and magnetic polarization *J*_p_ = 1 T for two different widths *w* = 5, 30 mm.

Sample Grade	Punching		*E*_P_	Water		*E*_w_
	*W*_tot-5mm_	*W*_tot-30mm_	[%]	*W*_tot-5mm_	*W*_tot-30mm_	[%]
M400-65A	53.041	39.541	25.452	36.636	31.297	14.573
M400-50A	42.909	32.658	23.890	25.671	22.429	12.629
M300-35A	29.292	19.869	32.169	21.865	18.771	14.150
NO20	20.992	15.566	25.847	15.951	15.024	5.811

Where *E* was computed as: (*W*_tot-5mm_ − *W*_tot-30mm_)/*W*_tot-5mm_ * 100.

**Table 4:** Average grain size <*s*> for the investigated non-oriented alloys.

Sample Grade	<*s*> [µm]
M400-65A	127
M400-50A	92
M300-35A	86
NO20	122
